# Treatment of Narcolepsy Type 1 With Orexin: A Systematic Review

**DOI:** 10.7759/cureus.76692

**Published:** 2024-12-31

**Authors:** Tania G Thomaz, Billy McBenedict, Dennys K Meireles, Giovanna F Farias, Luiz C Almeida, Marina C de Almeida Leitão, Wilhelmina N Hauwanga, Bruno Lima Pessôa, Maria Isabel do Nascimento

**Affiliations:** 1 Physiology and Pharmacology, Fluminense Federal University, Niterói, BRA; 2 Neurosurgery, Fluminense Federal University, Niterói, BRA; 3 Internal Medicine, Fluminense Federal University, Niterói, BRA; 4 Oncology, Fluminense Federal University, Niterói, BRA; 5 Cardiology, Gaffrée and Guinle University Hospital, Federal University of the State of Rio de Janeiro, Rio de Janeiro, BRA; 6 General and Specialized Surgery, Fluminense Federal University, Niterói, BRA

**Keywords:** cataplexy, excessive daytime sleepiness (eds), intranasal orexin treatment, narcolepsy type 1, orexin-a, rem sleep regulation

## Abstract

Narcolepsy is a rare, chronic neurological disorder characterized by excessive daytime sleepiness (EDS). Narcolepsy type 1 is probably caused by an autoimmune-mediated loss of orexin-producing neurons. Type 2 patients retain the physiological functioning of orexigenic neurons. The basis for treating narcolepsy type 1 with orexin-A is that if narcolepsy develops because of a loss of orexigenic neurons, then administering orexin should be able to eliminate, reduce, or prevent the impact of this loss. The aim of this review was to capture and analyze studies to elucidate the efficacy of orexin-A in the treatment of narcolepsy type 1 in humans. The search strategy included the following descriptors: “narcolepsy,” “orexin,” and “treatment,” with filters for randomized clinical trials (RCT) and human studies. A total of 70 publications were retrieved from the databases. Duplicate records were removed before screening (n = 13), and 54 were then excluded for the following reasons: off-topic (n = 18), reviews (n = 14), use of a different intervention other than orexin (n = 14), non-human studies (n = 4), out-of-population selection criteria (n = 2), and case report (n = 2). Thus, the studies included in the review were three. Treatment of narcolepsy with orexin decreases the number of wake-REM (rapid eye movement) transitions and total time of REM sleep, although it does not increase wake time. The failure of orexin to alleviate daytime sleepiness suggests that orexin deficiency is not the only factor involved in the pathophysiology of type I narcolepsy.

## Introduction and background

Narcolepsy is a rare, chronic neurological disorder characterized by excessive daytime sleepiness (EDS), with or without paralysis. Involuntary daytime sleepiness is the main symptom and can be accompanied by one or more of the following symptoms: hypnagogic and hypnopompic hallucinations, sleep paralysis, and cataplexy. A lifelong diagnosis of narcolepsy is usually made between the ages of 15 and 30 years, with a prevalence of about 1 per 3,000 people [1]. EDS and cataplexy are reported to be the common symptoms among narcolepsy patients [2].

Narcolepsy is divided into two subtypes: narcolepsy type 1 and narcolepsy type 2. Clinically, these subtypes are similar, except for cataplexy, which only occurs in patients with narcolepsy type 1. The diagnosis of narcolepsy is performed clinically using two specialized tests: Polysomnography (PSG) and the Multiple Sleep Latency Test (MSLT), which are supported by biomarker positivity for HLA-DQB106:02. Narcolepsy type 1 is probably caused by an autoimmune-mediated, significant, selective, and irreversible loss of hypocretin (orexin) neuropeptide-producing neurons. These orexigenic neurons synthesize and secrete both orexin‐A (hypocretin-1) and orexin‐B (hypocretin-2), and their loss leads to the orexin deficiency observed in type 1 narcolepsy. Type 2 narcolepsy patients retain the physiological functioning of orexigenic neurons, and therefore, orexin concentrations are normal. It is even less clear what causes narcolepsy type 2, since 10% to 20% of patients show a reduction in orexin, and 40% are positive for HLA-DQB106:02 [1].

Orexin-A and orexin-B (hypocretins) are neuropeptides synthesized in the lateral hypothalamus that bind to two specific G protein-coupled receptors, OX1R and OX2R [3,4]. OX1R has a high affinity for orexin-A and signals through Gq pathways, while OX2R binds both orexin-A and orexin-B with high affinity and can signal through Gq or Gi/Go pathways, depending on the target neuron [3,4]. These peptides play a critical role in promoting wakefulness by activating neural systems involved in arousal, including noradrenergic neurons in the locus coeruleus, histaminergic neurons in the tuberomammillary nucleus, serotonergic neurons in the raphe nuclei, and dopaminergic neurons in the ventral tegmental area [3,4]. Orexin neurons, responsive to diverse inputs related to stress, circadian rhythms, and motivation, project broadly across the central nervous system to maintain stable arousal and prolonged wakefulness, while integrating arousal, energy homeostasis, and reward mechanisms [3,4]. Mechanistically, orexins enhance neuronal excitability by inhibiting potassium channels, promoting calcium influx, and modulating the release of neurotransmitters such as glutamate and gamma-aminobutyric acid (GABA) at presynaptic terminals [4]. The loss of orexin-producing neurons, a defining feature in narcolepsy, disrupts the activity of monoaminergic and cholinergic nuclei, impairing sustained wakefulness, sleep stabilization, and transitions between sleep states [3,4].

The earliest works related to the potential treatment or solution to narcolepsy can be linked to a project that was funded by the Defense Advanced Research Projects Agency (DARPA) in 2007, aimed at the discovery of a drug that could eliminate or highly reduce sleepiness [5]. The sparked interest in funding this research was seemingly not for the use of orexin to treat narcolepsy, but for other operations, such as the military, since pilots flying long distances use drugs such as amphetamines, which may lead to addiction and generally have side effects, such as elevated blood pressure and mood swings. Orexin-A, a peptide that participates in sleep regulation and is released by specific neurons in the hypothalamus, has widespread receptors in the brain that allow it to activate areas affected by sleep and sleep deprivation [5,6]. The study by Deadwyler et al. [5] led to the development of a nasal spray containing orexin-A, which was reported to reverse the effects of sleep deprivation in monkeys, with equivalent results for cognitive tests when compared to well-rested monkeys.

The basis for treating narcolepsy with orexin-A is that if narcolepsy develops because of a loss of orexigenic neurons, then administering orexin should be able to eliminate, reduce, or prevent the impact of this loss (narcolepsy). In addition, orexin-A is a naturally occurring brain neurotransmitter that is known to increase muscle tone, induce arousal, and lessen/offset the effects of narcolepsy [7,8]. Despite marked advances in the field of neurobiology and pathophysiology, particularly that of narcolepsy type 1, the treatment is still limited to the management of symptoms (Table 1) [9]. Thus, the objective of this study was to capture and analyze studies that used orexin-A in humans for the treatment of narcolepsy type 1 and to elucidate its efficacy in the treatment of narcolepsy type 1.

**Table 1:** Current drugs and doses used for the treatment of narcolepsy. Table credit: Extracted from Barateau and Dauvilliers [10] EMA, European Medicines Agency; FDA, U.S. Food and Drug Administration; NA, Not available

Drug	Daily doses for adults	Indication	Class of evidence for use in childhood narcolepsy	Approval
Modafinil	100-400 mg	Sleepiness	No clinical trial	FDA, EMA
Armodafinil	100-250 mg	Sleepiness	No clinical trial	FDA
Methylphenidate	10-60 mg	Sleepiness	No clinical trial	FDA, EMA
Sodium oxybate	4.5-9 g	Sleepiness, cataplexy, disturbed nighttime sleep	Recent trial with class I evidence	FDA, EMA
Pitolisant	9-36 mg	Sleepiness, cataplexy	Ongoing international clinical trial	EMA, FDA (sleepiness)
Solriamfetol	75-150 mg	Sleepiness	NA	FDA; under review by the EMA
D-amphetamines	5-60 mg	Sleepiness	No clinical trial	FDA
Serotonin and norepinephrine-reuptake inhibitors: venlafaxine	37.5-300 mg	Cataplexy	No clinical trial	-

## Review

Materials and methods

The review was designed to answer the following question: is orexin-A effective in the treatment of narcolepsy type 1? The systematic review was formatted as a narrative synthesis and developed according to the criteria established by the instrument Preferred Reporting Items for Systematic Reviews and Meta-Analyses (PRISMA) [11].

Search Strategies

The review was conducted between May 1, 2023, and June 15, 2023, by consulting the following bibliographic databases: (i) Medical Literature Analysis and Retrieval System Online (MEDLINE/PUBMED), (ii) Embase, (iii) Scopus, (iv) Biblioteca Virtual em Saúde (BIREME), (v) Science, and (vi) Cochrane Library. The search strategy included the following descriptors: “narcolepsy,” “orexin,” and “treatment.” Table 2 summarizes the strategy that was applied to each bibliographic database.

**Table 2:** Search strategies used for databases.

Database	Search strategy
PubMed	(Narcolepsy/therapy [MeSH Terms]) AND (orexin [MeSH Terms] OR hypocretin [MeSH Terms]); Filters: Clinical Trial, Humans
Embase	('narcolepsy'/exp OR narcolepsy) AND ('orexin'/exp OR orexin) AND ('treatment'/exp OR treatment); Filters: AND 'human'/de AND 'randomized controlled trial'/de
Scopus	(KEY (narcolepsy AND therapy) AND (KEY (orexin OR hypocretin)) AND (LIMIT-TO (EXACTKEYWORD, “Humans”)) AND (LIMIT-TO (SRCTYPE, “J”))
BIREME	(ti:(orexin)) or (ti:(hypocretin)) AND (ti:(narcolepsy)) AND (treatment)
Science	[All: narcolepsy] AND [All: and orexin] AND [All: and therapy]
Cochrane	#1 (narcolepsy): ti, ab, kw AND (orexin): ti, ab, kw AND (therapy): ti, ab, kw; #2 (narcolepsy): ti, ab, kw AND (hypocretin): ti, ab, kw AND (therapy): ti, ab, kw; #3 (narcolepsy): ti, ab, kw AND (orexin): ti, ab, kw AND (treatment): ti, ab, kw; #4 (narcolepsy): ti, ab, kw AND (hypocretin): ti, ab, kw AND (treatment): ti, ab, kw

Eligibility Criteria

The main interest was to identify randomized clinical trials (RCTs) that used orexin-A as a treatment for narcolepsy type 1 in humans. To have a broad view of the topic, we did not restrict our search to orexin-A or narcolepsy type 1. We used, instead, the raw terms “narcolepsy” and “orexin” or “hypocretin.”

Data Extraction and Quality Assessment

Six researchers were responsible for study selection and data collection. A form for recording data was prepared, considering the main characteristics of the study and the questions of interest. Five researchers would look for consensus in the inclusion or exclusion of a study. In cases where there was a lack of consensus on the inclusion of studies, the sixth and most experienced researcher (T.G.T.) analyzed the study and made the decision on its inclusion or not. The extracted data included: (i) publication characteristics (authorship, year, country, study classification); (ii) studies that attended to the formulated PICO (Population, Intervention, Control, Outcomes) [12], which was: Population - adult narcoleptic patients without any endocrine disease, Intervention - orexin, Control - placebo or any other approved drug for the treatment, Outcomes - results of any test used to diagnose narcolepsy (Olfactory Performance Test, PSG, Sleepiness Scales, Wakefulness Maintenance Test). The quality of the studies was evaluated by pairs of researchers using the Mixed Methods Appraisal Tool (MMAT) version 2018 user guide [13]. Regarding ethical aspects, this work follows the recommendations for research involving human beings and is exempted from evaluation by the Research Ethics Committee because it is research conducted exclusively with scientific texts retrieved from the scientific literature.

Results

Search and Screening Outcomes

**Figure 1:**
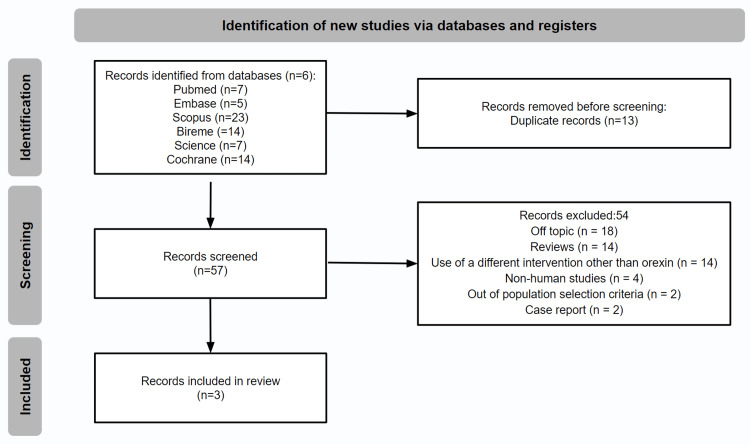
Flowchart of the steps that were performed in the systematic review for the identification, screening, and inclusion of the studies.

**Figure 2:**
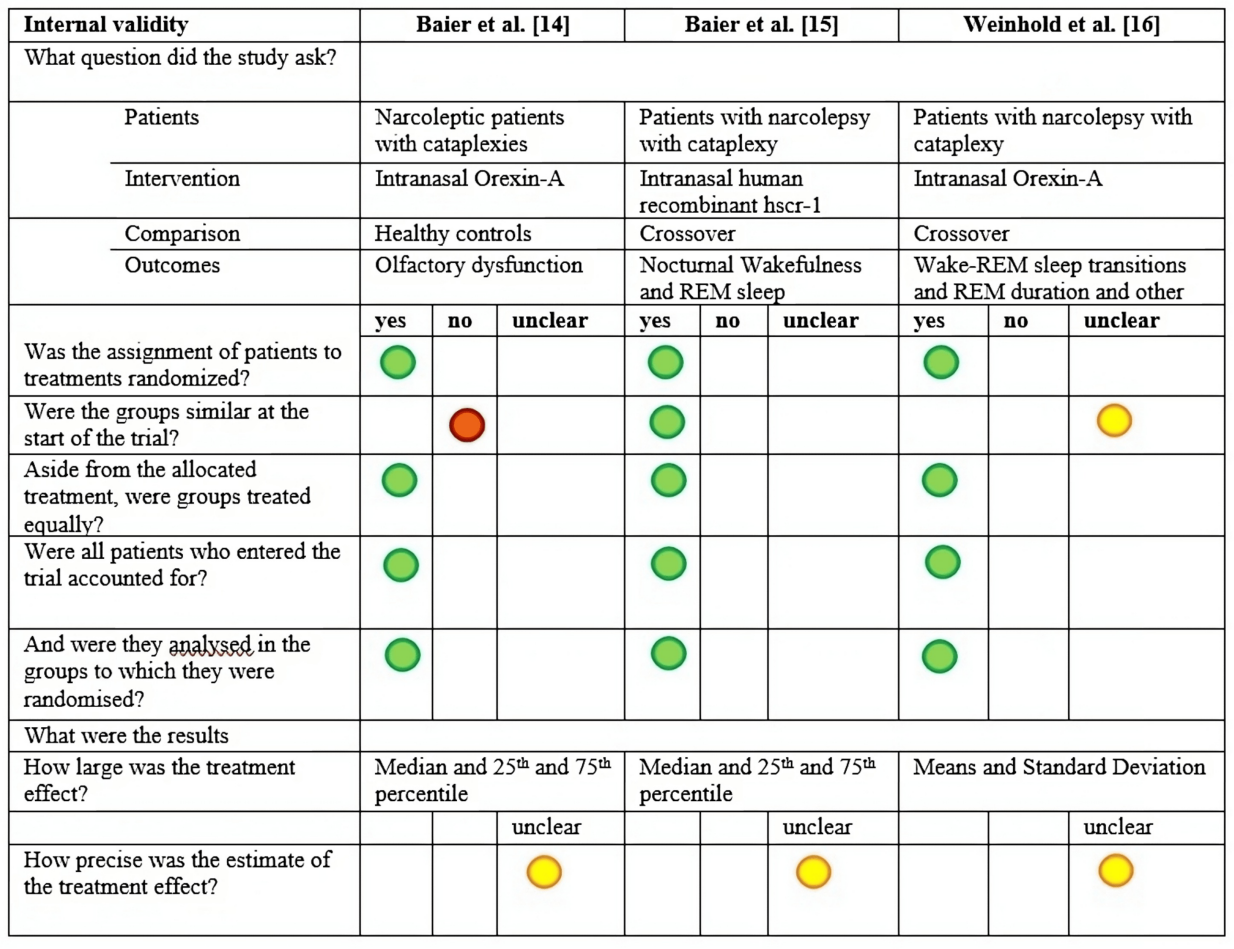
Quality assessment of the studies selected and included in this systematic review. Image credit: Adapted from the Mixed Methods Appraisal Tool (MMAT) version 2018 user guide. Available from: http://mixedmethodsappraisaltoolpublic.pbworks.com/w/file/fetch/146002140/MMAT_2018_criteria-manual_2018-08-08c.pdf REM, Rapid eye movement

A total of 70 publications were retrieved, and after the exclusion of 13 duplicate records, 57 records were screened through title and abstracts. Despite the extensive literature on the role of orexin in type I narcolepsy, only three articles met our inclusion criteria and PICO statements (Figure 1). The three studies included in the review were assessed for quality using the selected tool, and the results are shown in Figure 2.

Bias Assessment and Study Quality Evaluation

The evaluation began with two screening questions to ensure that each included study collected data relevant to its research questions. Studies that did not meet these criteria were excluded. Each eligible study was then classified into one of five methodological categories: qualitative, quantitative RCTs, quantitative non-randomized studies, quantitative descriptive studies, or mixed methods. For each category, five specific quality criteria were assessed, including aspects such as randomization, outcome completeness, blinding, representativeness, measurement validity, and fidelity of intervention administration, depending on the study type. Each criterion was rated as "Yes," "No," or "Can’t tell," with efforts made to address unclear information by consulting additional documentation. Importantly, the MMAT discourages assigning an overall score, instead emphasizing detailed criterion-level reporting to provide a nuanced understanding of study quality. To minimize bias, independent assessments were conducted by multiple reviewers, and any discrepancies were resolved through discussion or adjudication by an experienced reviewer. Despite the robust assessment framework, the inclusion of only three RCTs with small sample sizes posed a limitation to the review’s overall conclusions. This systematic approach ensured a transparent and thorough evaluation of study quality and bias for the included studies.

Characteristics of Included Studies

The three RCTs that used orexin-A for the treatment of type I narcolepsy are from the same research group at the University of Kiel in Germany (Table 3). None of the studies reported any incidents or undesirable side effects using orexin-A. The first RCT that explored the use of orexin in type I narcolepsy seems to be from 2008 [14]. This RCT was published a year after the study by Deadwyler et al. [5], which demonstrated that orexin eliminated the effects of sleep deprivation in monkeys. However, Baier et al. [14] did not evaluate the effects of orexin on daytime sleepiness, typical of narcoleptics, but evaluated olfactory dysfunction, which is also characteristic of narcoleptics. In this case, orexin improved olfactory function when administered to type I narcoleptic patients.

A second RCT was published by the same group [15], three years later, with the aim of evaluating the effect of orexin during night sleep in type I narcoleptic patients using PSG. Orexin was administered as a nasal spray at 10 pm. It is known that the sleep architecture of narcoleptics is deeply compromised, with excessive time in rapid eye movement (REM) sleep, abrupt transitions between wakefulness and REM sleep, and a longer wakefulness period during the night. However, what bothers narcoleptic patients the most is EDS. In this sense, the study’s strategy of administering orexin at night, before the period of nocturnal sleep, aimed to investigate the function of orexin not in maintaining daytime wakefulness, but rather in the possibility of inducing sleep with physiological characteristics - that is, of better quality.

In fact, this study showed that night sleep was closer to physiological sleep in narcoleptics after the administration of orexin at night. Total time in REM sleep decreased, as well as abrupt sleep-wake transitions. On the other hand, the time spent awake during the night did not decrease with the use of orexin. Considering that the function of maintaining wakefulness is attributed to orexin, and type I narcolepsy is precisely characterized by the lack of orexin and the inability to maintain wakefulness during the day, it is not at all unexpected that study participants stayed awake for a longer time during the night, since the orexin was administered at 10 pm. Finally, this work evaluated daytime sleepiness using the Stanford Sleepiness Scale (SSS) and did not detect any modifications after administering orexin the night before. Orexin did not increase daytime wakefulness the following day when administered the night before.

Coherently, the same group published a third RCT, again three years later, in which they administered orexin in the morning at 7 am [16]. In addition to the SSS and PSG, which were performed on the night of the day of orexin administration, the authors also evaluated the patient's daytime naps using the modified Maintenance of Wakefulness Test, similar to the MSLT, and a divided attention test. There is an undeniable expectation that administering orexin immediately after waking up would relieve daytime sleepiness, which highly impairs the quality of life of narcoleptic patients. However, once again, when orexin was given in the morning, it was unable to satisfactorily maintain wakefulness during the day. Patients continued to fall asleep during the day, and the total awake time during the day was not altered by orexin. There was no difference in the SSS with regard to comparing orexin to the placebo. On the other hand, the total time in REM sleep and the number of transitions from wakefulness to REM sleep during daytime naps decreased.

The attention test performed during the day showed fewer false reactions and a tendency towards a reduction in reaction time with the use of orexin. The authors of the study questioned whether the observed effect of improved attention was due to orexin or a consequence of improved sleep quality during daytime naps. The orexin administered in the morning less intensely affected the night sleep that day. PSG, performed on the night of the day orexin was administered, showed a tendency towards a reduction in the number of wake-REM transitions and an increase in the duration of phase 2 of non-REM sleep - that is, a smaller effect than was observed when orexin was administered at 10 pm. The less relevant effect of this daytime administration of orexin probably reflects the drop in orexin concentration after its administration in the morning. The data from the aforementioned studies were tabulated and presented in Table 3, which describes the included articles and their main information. The information was outlined using the PICO strategy.

**Table 3:** Characteristics and main findings of the studies included in the systematic review. ^1^: PEA, 2-phenyl-ethyl alcohol; ^2^: REM, Rapid eye movement; ^3^: N2 e N3, Non-rapid eye movement sleep stages

Reference	Population	Intervention	Control	Outcomes	Results
Baier et al. [14]	7 women, 3 men, adults (24 to 67 years) with type 1 narcolepsy	Olfactory Performance Test	Healthy adults	Threshold, discrimination, and identification of items in the Olfactory Performance Test	Narcoleptics performed worse when compared to healthy controls
	5 women, 2 men adults (24 to 67 years) with type 1 narcolepsy	0.1 mL intranasal orexin-A at 11 pm in each nostril, at 1-minute intervals for 10 minutes, with an approximate total volume of 2 mL	Placebo	PEA¹ threshold detection test. The test was performed at the beginning of the study, after the use of a placebo, and after the use of orexin	Orexin-A improved the thresholds of detection of PEA¹ compared to placebo
Baier et al. [15]	5 women, 3 men, adults (24 to 67 years) with type 1 narcolepsy	0.1 mL intranasal orexin-A at 10 p.m. in each nostril, at 1-minute intervals for 10 minutes, with an approximate total volume of 2 mL	Placebo	Polysomnography	Awake time - orexin did not change the awake time during night sleep. Time in REM² sleep - orexin decreased time in REM² sleep in the second half of night sleep, thus decreasing total REM² sleep time. Wake-REM² transitions - orexin reduced the amount of wake-REM² transitions, thereby stabilizing sleep.
				Stanford Sleepiness Scale	# There was no difference in the sleepiness scale scores when compared to placebo
Weinhold et al. [16]	8 women, 6 men, adults (18 to 64 years) with type 1 narcolepsy	0.1 mL intranasal orexin-A at 7 a.m. in each nostril, at 1-minute intervals for 10 minutes, with an approximate total volume of 2 mL	Placebo	Modified Wakefulness Maintenance Test	# During daytime naps: Awake time - orexin did not change the awake time during the day. Time in REM² sleep - orexin decreased time in REM² sleep. Wake-REM² transitions - orexin reduced the amount of wake-REM² transitions, thereby stabilizing sleep
				Polysomnography	Night sleep after day of test: increased duration of N2³ with the use of orexin tendency to reduce the number of Wake-REM² transitions; reduced N3³ latency
				Divided Attention Test	Fewer false reactions and a tendency to reduce reaction time with the use of orexin
				Stanford Sleepiness Scale	There was no difference in the sleepiness scale scores when compared to placebo

Discussion

Effectiveness of Orexin-A in REM Sleep Stabilization

It was surprising to find only three RCTs that used orexin as a treatment for narcolepsy, especially when none of them reported any incident or undesirable side effects. Despite the reduced number of patients, which is justifiable in the case of type I narcolepsy, the study design of the last RCT presented a viable application of orexin. Although none of the RCTs showed changes in the length of daytime wakefulness, the use of orexin proved to be promising in recovering sleep physiology and improving attention.

Baier et al. [14] study highlighted the significant differences in olfactory performance between narcolepsy patients with cataplexy and matched healthy controls, with the administration of intranasal orexin-A showing a statistically significant improvement in olfactory thresholds (p = 0.016). While the statistical analyses performed - such as the Mann-Whitney U-test and Wilcoxon Signed Rank test - demonstrate the validity of these findings, further exploration of the clinical relevance of these results is warranted. According to Baier et al. [14], the restoration of olfactory function through orexin-A administration, as evidenced by increases in olfactory performance metrics such as the TDI (Threshold Detection Index) score and PEA (2-phenyl-ethyl alcohol) thresholds, provides compelling support for the role of orexin deficiency in narcolepsy-associated olfactory dysfunction. However, the study did not systematically assess whether these improvements translate into meaningful benefits in patients' daily lives or broader functional outcomes [14]. 

Baier et al. [15] showed promising findings regarding the intranasal administration of orexin-A (hypocretin-1) in narcolepsy patients with cataplexy, demonstrating both significant statistical outcomes and clinical potential. Specifically, orexin-A significantly restored olfactory dysfunction (p < 0.05) and reduced REM sleep instability (p = 0.02) in controlled settings [15]. These findings demonstrated the role of orexinergic pathways in the physiological and symptomatic aspects of narcolepsy. However, the interpretation of the clinical relevance of these results requires a deeper discussion. While statistical significance confirms the reliability of the findings, effect sizes and confidence intervals should be included to evaluate the magnitude of these changes more comprehensively. For instance, the improvement in olfactory thresholds and REM sleep stabilization suggests potential quality-of-life benefits for patients [15]. Yet, the practical implications of these outcomes, such as their influence on daily functioning, alertness, and symptom severity, were not explored in detail. The absence of long-term follow-up data also limits the ability to assess sustained therapeutic impact.

Weinhold et al. [16] study demonstrated that intranasal administration of orexin-A in patients with narcolepsy with cataplexy significantly reduced daytime REM sleep duration (p = 0.041) and wake-REM sleep transitions (p = 0.022). These results indicate the potential of orexin-A as a REM sleep-stabilizing agent, supporting its role in addressing the fragmented sleep patterns characteristic of narcolepsy. The study also observed enhanced cognitive performance, with fewer false reactions in a divided attention task (p = 0.026), suggesting improvements in neuropsychological functions following orexin-A administration [16]. Despite these promising outcomes, further discussion is necessary to contextualize the clinical relevance of these findings. While the reduction in REM sleep instability and improved attention are encouraging, their direct impact on patient quality of life and functional outcomes was not fully explored. Additionally, the long-term benefits of intranasal orexin-A administration remain to be determined, as the study focused on the effects of a single dose [16].

We believe it is crucial to further investigate the potential benefits of orexin-A, particularly given the limited number of RCTs currently available. However, the scientific community has largely shifted its focus from evaluating exogenous orexin-A to developing receptor-targeted drugs, such as orexin agonists and histamine receptor inverse agonists, to modulate these pathways more effectively [17-20]. The disappointing outcome of orexin's inability to alleviate daytime sleepiness underscores that the role of orexin in maintaining wakefulness remains poorly understood. Given that narcolepsy stems from hypothalamic dysregulation, it is plausible that the condition manifests with varying degrees of intensity or severity. This variability likely arises from the intricate network of interactions within the hypothalamus, reflecting the complexity of its regulatory mechanisms [7,21-23]. An explanation for orexin's inability to alleviate daytime sleepiness could be that the immune reaction that destroys orexin-producing neurons in the lateral hypothalamus also affects orexin receptors [1]. However, several animal studies showed significant improvement in all symptoms of narcolepsy after administration of exogenous orexin [24,25]. Other studies have demonstrated improvement in humans with the use of orexin receptor agonists, and histamine H3 receptor agonists have also been shown to provide relief of narcolepsy symptoms in animals and humans [17,19].

Orexin Deficiency and Neural Pathways in Narcolepsy

Type II narcolepsy clinically resembles type I narcolepsy in EDS but lacks cataplexy or orexin deficiency [26]. This is in line with the fact that the administration of orexin does not reverse daytime sleepiness in patients with orexin deficiency. If orexin deficiency were solely responsible for daytime sleepiness, how can we explain the same daytime sleepiness in patients without orexin deficiency? The first function attributed to orexin was the modulation of eating behavior. The discovery of orexin deficiency in type I narcoleptic patients was also consistent with the role of orexin in the modulation of the sleep-wake cycle, since an animal needs to be awake in order to look for food [27]. The hypothesis that orexin deficiency caused type I narcolepsy was consolidated in the late 1990s. In fact, cerebrospinal fluid orexin levels in animal and human models of narcolepsy are reduced, which led to the distinction between type I and type II narcolepsy [2,28-30].

The fact that the administration of orexin is able to improve sleep quality, principally the physiology of REM sleep, but is not able to increase wakefulness suggests that orexin deficiency may not be the only factor responsible for type I narcolepsy [21,26,31]. A question not addressed by any of the three RCTs is whether orexin could reduce episodes of cataplexy and sleep paralysis, phenomena that are probably caused by orexin deficiency. In narcoleptic patients, positive emotions, such as laughter, can provoke an episode of cataplexy. Sudden, yet positive, emotions activate neurons in the medial prefrontal cortex that excite orexin neurons in the lateral hypothalamus and the central amygdala. The absence of orexin, produced in the lateral hypothalamus, leads to reduced activity of GABAergic neurons in the periaqueductal grey area that inhibits rapid eye REM sleep and increases the activity of glutamatergic neurons in the sublaterodorsal tegmental nucleus (pons), which are involved in REM atonia. The imbalance in this pathway results in the activation of descending pathways that inhibit spinal motor neurons, eventually leading to cataplexy [32].

Sleep paralysis, a phenomenon that can occur in healthy individuals but is more prominent in individuals with narcolepsy, arises during the transition from REM sleep to wakefulness [33]. It represents a disruption in the normal mechanisms governing the shift to wakefulness, where the individual regains consciousness but remains temporarily unable to move their muscles. This paralysis is caused by the persistent activation of neurons in the sublaterodorsal tegmental nucleus (located in the pons), which form part of the reticulospinal tract [33]. These neurons typically function to suppress motor activity during REM sleep, preventing body movements, but their continued activation during wakefulness results in the temporary inability to move [33].

The fact that intense emotions, especially laughter, cause muscle weakness has been reported in approximately 15% of healthy individuals. Overeem et al. [34] recorded muscle weakness caused by laughter using the H-reflex test in electroneuromyography. In the study, all tested healthy individuals showed abolition or a decrease of the H-reflex when listening to jokes that made them laugh. It is possible to conclude that laughter causes cataplexy in narcoleptic patients and varying degrees of hypotonia in healthy individuals, depending on the amount of orexin available. Healthy individuals genetically predisposed to lower production of orexin would experience more intense and frequent episodes of hypotonia, while individuals with neuronal loss of orexin-producing neurons (type I narcoleptics) would present the so-called cataplexy.

The hypothesized lack of increased wakefulness with orexin-A administration can be examined in light of recent insights into the plasticity and regulatory complexity of the orexinergic system. Recent studies demonstrate that the orexinergic system exhibits dynamic neural plasticity, adapting to both short-term and chronic perturbations in signaling pathways. For instance, research on suvorexant treatment in animal models revealed autoregulatory changes in orexin receptor expression, supporting the notion of a tightly controlled homeostatic mechanism within this system [35,36]. Furthermore, orexin neurons are known to undergo changes in synaptic connectivity and receptor activity, depending on physiological states such as sleep-wake transitions and metabolic demands [35,37]. This adaptive plasticity could explain the limited efficacy of exogenous orexin-A in directly modulating wakefulness in conditions like narcolepsy type 1, where a significant loss of orexin neurons disrupts the network's ability to coordinate wake-promoting circuits effectively [38]. Additionally, interactions with complementary systems, such as the histaminergic pathways, have been highlighted. Pharmacological studies show that enhancing histaminergic signaling via H3-receptor inverse agonists can partially compensate for orexin deficits, further underscoring the interplay between these systems in regulating arousal​ [17]. Thus, the limited impact of orexin-A on wakefulness may arise from its dependence on a broader functional network, which is already compromised in narcolepsy due to neuronal loss and impaired plasticity. Future studies exploring combination therapies targeting multiple pathways, including histaminergic and orexinergic systems, may provide more effective solutions for managing the disorder.

Recommendations

To build on the findings of this study, future research should prioritize investigating the long-term effects of intranasal orexin-A administration on olfactory function and its broader impact on symptoms of narcolepsy, including sleep regulation and cataplexy. Longitudinal studies are essential to determine whether the observed improvements in olfactory performance, REM sleep stability, and attention are sustained over time, and how they translate to real-world benefits for patients. In addition, future studies should incorporate effect size measurements and confidence intervals to provide a detailed understanding of the statistical and clinical significance of these findings.

Exploring dose-response relationships and optimizing administration schedules will be critical to identifying the most effective and practical use of intranasal orexin-A. Broader investigations into how these findings affect the management of narcolepsy, particularly improvements in EDS and the frequency and severity of cataplexy, are also necessary to fully realize the therapeutic potential of orexin-A. By combining rigorous statistical analyses with comprehensive clinical evaluations, future research can bridge the gap between experimental findings and their application in clinical practice. 

Limitations of the review

This is a systematic review, whose protocol was not registered at the International Prospective Register of Systematic Reviews (PROSPERO). We consulted PROSPERO in September 2023, and there was no protocol for such a review. The inclusion of only three RCTs, with a reduced number of patients, makes it difficult to draw strong conclusions on the efficacy of orexin in the treatment of narcolepsy.

## Conclusions

Treatment of narcolepsy with orexin decreases the number of wake-REM transitions and total time of REM sleep, although it does not increase wake time. Orexin, despite not being able to increase wakefulness in narcoleptic patients, is able to transform the patient's sleep into a more physiological sleep. The failure of orexin to alleviate daytime sleepiness suggests that orexin deficiency is not the only factor involved in the pathophysiology of type I narcolepsy. The three RCTs included in this review do not provide insights to explain the failure to successfully use orexin to reverse the main symptoms of type I narcolepsy in humans, despite the promising results found in animal models. The findings call for a paradigm shift toward combination therapies that address multiple neurobiological pathways, including histaminergic and orexinergic systems, to achieve more comprehensive management of NT1 symptoms. Future research should focus on long-term studies assessing the sustained impact of orexin-A and its integration with adjunctive pharmacological approaches to optimize treatment strategies.
